# Receipt of Opioid Agonist Treatment in provincial correctional facilities in British Columbia is associated with a reduced hazard of nonfatal overdose in the month following release

**DOI:** 10.1371/journal.pone.0306075

**Published:** 2024-07-10

**Authors:** Katherine E. McLeod, Jane A. Buxton, Mohammad Ehsanul Karim, Ruth Elwood Martin, Bohdan Nosyk, Megan Kurz, Marnie Scow, Guy Felicella, Amanda K. Slaunwhite

**Affiliations:** 1 Department of Family Medicine, McMaster University, Hamilton, Canada; 2 British Columbia Centre for Disease Control, Vancouver, British Columbia, Canada; 3 Faculty of Medicine, School of Population and Public Health, University of British Columbia, Vancouver, British Columbia, Canada; 4 Centre for Health Evaluation and Outcome Sciences (CHÉOS), St. Paul’s Hospital, Vancouver, British Columbia, Canada; 5 Health Economic Research Unit, British Columbia Centre for Excellence in HIV/AIDS, Vancouver, British Columbia, Canada; 6 Faculty of Health Sciences, Simon Fraser University, Burnaby, British Columbia, Canada; 7 British Columbia Centre on Substance Use, Vancouver, British Columbia, Canada; Medizinische Universitat Wien, AUSTRIA

## Abstract

**Background:**

In many jurisdictions, policies restrict access to Opioid Agonist Treatment (OAT) in correctional facilities. Receipt of OAT during incarceration is associated with reduced risk of fatal overdose after release but little is known about the effect on nonfatal overdose. This study aimed to examine the association between OAT use during incarceration and nonfatal overdose in the 30 days following release.

**Methods and findings:**

Using linked administrative healthcare and corrections data for a random sample of 20% of residents of British Columbia, Canada we examined releases from provincial correctional facilities between January 1, 2015 –December 1, 2018, among adults (aged 18 or older at the time of release) with Opioid Use Disorder. We fit Andersen-Gill models to examine the association between receipt of OAT in custody and the hazard of nonfatal following release. We conducted secondary analyses to examine the association among people continuing treatment initiated prior to their arrest and people who initiated a new episode of OAT in custody separately. We also conducted sex-based subgroup analyses. In this study there were 4,738 releases of 1,535 people with Opioid Use Disorder. In adjusted analysis, receipt of OAT in custody was associated with a reduced hazard of nonfatal overdose (aHR 0.55, 95% CI 0.41, 0.74). This was found for prescriptions continued from community (aHR 0.49, 95%CI 0.36, 0.67) and for episodes of OAT initiated in custody (aHR 0.58, 95%CI 0.41, 0.82). The effect was greater among women than men.

**Conclusions:**

OAT receipt during incarceration is associated with a reduced hazard of nonfatal overdose after release. Policies to expand access to OAT in correctional facilities, including initiating treatment, may help reduce harms related to nonfatal overdose in the weeks following release. Differences in the effect seen among women and men indicate a need for gender-responsive policies and programming.

## Introduction

Criminal legal systems around the world incarcerate a large number of people who use drugs, including people with Opioid Use Disorder (OUD) [1] Opioid Agonist Treatment (OAT) are medications, such as buprenorphine/naloxone and methadone, used to treat OUD. Access to OAT in custody varies by jurisdiction and is rarely equivalent to access in the community [2–4]. Incarceration is a common reason for interruption of OAT [3, 5, 6]. There has been limited research on the impact of OAT dispensed in custody. We aim to address some of the existing gaps in the literature by exploring the impact of OAT prescribed in custody on nonfatal overdose after release with specific attention to the effects of gender and differences in recent history of OAT use.

Nonfatal overdoses occur at 10–50 times [7–9] the rate of fatal overdose. Nonfatal overdose is a risk factor for subsequent fatal overdose [10] and is associated with significant acute and long-term morbidity [11, 12] Studies have shown that release from custody is a period of elevated risk for fatal overdose [13–18] but the literature on nonfatal overdose is inconsistent. A 2019 meta-analysis of studies examining people who inject drugs did not find an association between recent incarceration and nonfatal overdose, [19] though cohort studies in the US, [20] Australia [21] and Canada [22] have shown elevated incidence of nonfatal overdose in the four weeks following release from custody. Studies have found that continuity of OAT in custody is protective against all-cause mortality and fatal overdose after release, [23–25] but there is little research examining the potential effect of OAT prescribed in custody on nonfatal overdose after release [26]. There are complex intersecting, individual, structural and environmental factors that influence risk of overdose and whether an overdose event is fatal [27, 28]. Evidence specific to nonfatal overdose is needed to guide comprehensive and impactful response to the effects of the toxic drug supply.

Additionally because in many jurisdictions OAT is not available in custody or is provided only to people who have an active prescription when they are admitted [2–4] existing literature on OAT in correctional settings has predominantly focused on people who were using OAT in the community at the time of their incarceration and compares outcomes for people who had their OAT prescriptions continued in custody and people who were discontinued [3, 4, 23, 24, 26]. In British Columbia (BC) recent policy changes expanded access to OAT in provincial correctional facilities, particularly for people who are not actively using OAT when they are admitted [29]. Incarceration may be a unique opportunity to offer treatment and services because people often have fewer priorities competing with healthcare needs (such as finding housing or employment) compared to when they are in the community [30]. While the potential to initiate people into treatment exists, interruptions in care during the transition between prison and community are common, indicating a need for thoughtful inquiry into the potential benefits and risks.

Intersecting structural and social factors shape experiences and risks of incarceration, substance use, treatments, and overdose differently for women and men. For example, women experience greater stigma related to drug use and are more vulnerable to consequences for disclosing use or seeking treatment including homelessness, violence and having their children apprehended [31]. The prevalence of substance use disorder is higher among incarcerated women than among men in custody or non-incarcerated women [1, 32]. Women also spend shorter periods of time incarcerated, [33] which may affect access to services. Though evidence is mixed, studies suggest there may be a relationship between sex and overdose risk. Cohort studies in the US [14, 34] and Norway [35] found increased risk of fatal overdose after release among women. However, another US cohort study found an increased risk of overdose mortality among men in the first two weeks after release but no difference in subsequent weeks [36]. Other studies have not found a relationship [13, 17, 34]. To inform appropriate policy and action there is a need to understand sex and gender differences in access to OAT in carceral settings and the potential impact of OAT on overdose after release.

The primary aim of this study was to examine the relationship between receipt of OAT while incarcerated and the hazard of nonfatal overdose among people who have OUD during the four-week period following release when risk of overdose is highest [14, 15, 21, 37]. The second aim was to assess this relationship among people who are initiating a new episode of OAT in custody and people continuing a community prescription separately. As a third aim, we examined the relationship between receipt of OAT during incarceration and nonfatal overdose after release among women and men separately.

## Methods

### Data source and study design

**Fig 1:**
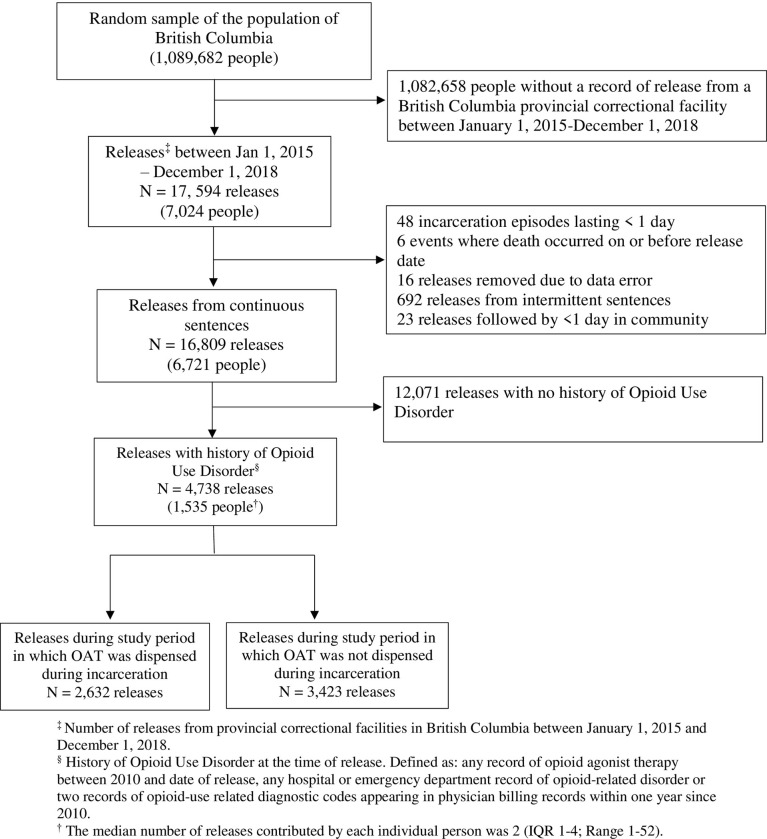
Flow chart of the analytic sample selection using data from the 20% random sample of British Columbia population included in the Provincial Overdose Cohort for releases from provincial correctional facilities between January 1, 2015—December 1, 2018.

We conducted a retrospective cohort study using linked administrative data from the BC Provincial Overdose Cohort [38]. Healthcare, pharmaceutical and criminal-legal data (S1 Table) are linked using name, birthdate and the lifetime ten-digit personal health number assigned to each resident of BC as part of the universal health insurance program. The Provincial Overdose Cohort includes a representative 20% random sample (approximately 1.1 million people) of the BC population. Within this random sample, we included all releases from BC provincial correctional facilities between January 1, 2015, and December 1, 2018, among people with OUD and aged 18 and over at the time of release (Fig 1). Provincial correctional facilities hold people who have been sentenced to less than two years in custody or who have been remanded to custody to wait for trial or sentencing. OUD was defined as having at least one OAT dispensation between 2010 and the day of release, a hospital or emergency department record related to OUD or two diagnostic codes in physician billing records related to OUD within one year between 2010 and the date of release (S2 Table). Each release was counted separately, so individual people could contribute multiple releases to the cohort. For each release, follow-up began on the day of release from custody and was censored at the first of reincarceration, death or 30 days. We excluded incarceration events lasting less than one day and releases where people spent less than one day in community. We also excluded intermittent sentences. Intermittent sentences are sentences of less than 90 days in which people serve most of their time in the community under conditions of parole but spend some time (usually weekends) in custody. Data was first accessed for this study on December 1, 2020. Ethics approval for this study was granted by the University of British Columbia Behavioral Research Ethics Board (H19-03731).

#### Primary exposure

Our primary exposure was a dichotomous variable (yes/no) of any OAT dispensation during incarceration. An incarceration episode which included dispensation of OAT on or after the date of admission and prior to the date of release was considered receipt of OAT during incarceration. We used records in the BC PharmaNet database (provincial prescription dispensations) to identify OAT use in community and in provincial correctional facilities. We defined active community treatment as a community prescription current within the six days prior to the admission date.

#### Outcome variable

The primary outcome of interest was experience of any nonfatal overdose in the 30 days following release (yes/no). This timeframe was based on a robust body of literature demonstrating that risk of overdose is significantly elevated up to 4 weeks following release [18, 22, 34, 35]. Nonfatal overdoses were identified using linked administrative data from ambulance, poison control, emergency department, hospital and physician billing records [38, 39]. A description of the datasets and the case definition of overdose used in each are provided in S1 and S2 Tables. To prevent over-counting, healthcare records less than one day apart (within two calendar days) were collapsed into a single overdose event.

#### Potential confounders and risk factors

From the literature, we identified demographic, [10, 13, 14, 34–36, 40, 41] health [10, 15, 21, 36, 40, 42–45] and incarceration [21, 26, 34–36, 40] factors known to be associated with overdose after release. These factors included: age (18–29, 30–49, 50 and older), sex (female or male; no other category was present in the data), number of days of most recent incarceration (1–4 days, 5–15 days, 16–52 days, >53 days; categorized based on the distribution of the data), and number of previous provincial incarcerations since 2010 (0,1, 2+). We used year of release to account for changes in risk of overdose over time due to increasing presence of fentanyl and its analogs in the illicit drug supply [46]. We also included mental health condition (yes/no) and number of chronic health conditions (0, 1+) diagnosed between 2010 and date of release. When healthcare services in BC’s provincial correctional facilities transferred from a private contractor to the Ministry of Health on October 1, 2017 access to OAT was made a key priority [47, 48]. Therefore, in sensitivity analyses, we also examined whether release occurred before or after the transfer. Death, including those from fatal overdose were identified using data from the BC Coroners Service and Vital Statistics.

### Statistical analyses

We used the chi-square test to compare characteristics of releases in which the person received OAT in custody with those that did not. For all analyses we applied Andersen-Gill [49] regression models with robust error variance [50]. The Andersen-Gill model is a generalization of the Cox proportional hazards model [51]. It allows for examination of recurrent event data which follows a Poisson process [52]. This means that the model accounted for an individual person experiencing multiple nonfatal overdose events during follow-up. It is not well understood how experiencing a nonfatal overdose may affect the timing or risk of a subsequent nonfatal overdose. A small number of studies have suggested that those who have previously experienced a nonfatal overdose at some point in their history may be at an elevated risk for another nonfatal overdose [21, 44, 45]. However, we could not find any study to suggest that such relationships persist in a short period of follow up (e.g., 30 days) which was the interest of this study. Since there is no known or obvious biological mechanism that might create a relationship between the timing of overdose events, the primary assumption was that the risk of nonfatal overdose remains constant during the 30 days of follow-up and does not depend on the number of previous overdose events in the preceding days or weeks. For this reason, and also to avoid adjusting for post-baseline factors that might lead to over-adjustment due to potential adjustment of mediator or collider [53] we did not include or adjust for a time-dependent covariate counting the number of events during follow-up. We used a robust error variance as proposed by Lin and Wei [50] to account for correlation between events among individuals in the study [54]. For the primary analysis we examined the association between any use of OAT during incarceration and any nonfatal overdose in the 30 days following release. As a sensitivity analysis of the primary aim, we used a more conservative definition of OAT use by counting only prescriptions dispensed between the date of admission and the date of release (excluding dispensations on the day of admission which were not distinguished from community dispensations prior to arrest). As a second sensitivity analysis, we examined the relationship between receipt of OAT in custody and any overdose (fatal and nonfatal) after release. We also conducted an analysis using release before or after the transfer of healthcare services to the Ministry of Health in place of year of release. Finally, we conducted sensitivity analyses negative binomial regression using robust standard error and offsets for variation in follow-up time between releases in this study. To address the second aim, we examined the association between OAT use in custody and nonfatal overdose after release among people who were initiating a new episode of OAT while in custody and those who were continuing community prescriptions separately. As a sensitivity analysis for the second aim, we examined the relationship between OAT use during incarceration and nonfatal overdose after release separately for people with and without any previous use OAT in community or custody who initiated a new episode in custody. As a third aim, we conducted a sex-based subgroup analysis using stratification. We used an interaction model as a sensitivity analysis for the third aim. Statistical Analyses were performed using SAS Enterprise Guide 7.1. We considered p-value <0.05 to be significant.

## Results

In this study, 1,535 people contributed a total of 4,738 incarceration episodes with a median of two releases (IQR 1–4) per person. Of the 25 deaths during follow-up 22 (88%) were fatal overdoses. There were 453 nonfatal overdose events recorded during follow up. OAT was dispensed in custody in 55.74% of incarceration episodes; 55.66% of OAT dispensed was buprenorphine/naloxone, 44.26% was methadone, less than one percent was morphine. A higher proportion of people who received OAT during incarceration were incarcerated for longer, had no chronic conditions, did not have a mental health diagnosis, were released after the transfer, and had a history of OAT use. Reincarceration was the reason for censoring in 21% of all releases. A similar proportion of men (56.09%) and women (53.42%) were dispensed OAT while in custody (Table 1).

**Table 1:** Characteristics of men and women at each release from provincial correctional facilities by receipt of OAT during incarceration between January 1, 2015, and December 1, 2018, in a 20% random sample of the population of British Columbia.

	Study Cohort N = 4738			Women N = 614			Men N = 4124		
	Did not receive OAT during incarceration N (%)	Received OAT while incarcerated N (%)	p-value^‡^	Did not receive OAT during incarceration N (%)	Received OAT while incarcerated N (%)	p-value^‡^	Did not receive OAT during incarceration N (%)	Received OAT while incarcerated N (%)	p-value^‡^
	2097 (44.26)	2641 (55.74)		286 (46.58)	328 (53.42)		1811 (43.91)	2313 (56.09)	
**Any overdose during follow-up** ^**†**^			<0.01			0.0706			<0.01
No	1892 (90.22)	2470 (93.53)		254 (88.81)	305 (92.99)		1638 (90.45)	2165 (93.60)	
Yes	205 (9.78)	171 (6.47)		32 (11.19)	23 (7.01)		173 (9.55)	148 (6.40)	
**Censored due to reincarceration**			<0.01			<0.01			<0.01
No	1532 (73.06)	2192 (83.00)		230 (80.42)	298 (90.85)		1302 (71.89)	1894 (81.88)	
Yes	565 (26.94)	449 (17.00)		56 (19.58)	30 (9.15)		509 (28.11)	419 (18.12)	
**Active community prescription**			<0.01			<0.01			<0.01
No	1953 (93.13)	1581 (59.86)		264 (92.31)	194 (59.15)		1689 (93.26)	1385 (59.88)	
Yes	144 (6.87)	1060 (40.14)		22 (7.69)	134 (40.85)		122 (6.74)	928 (40.12)	
**History of OAT use prior to incarceration** ^◊^			<0.01			<0.01			<0.01
No	600 (28.61)	465 (17.61)		79 (27.62)	52 (15.85)		521 (28.77)	413 (17.86)	
Yes	1497 (71.39)	2176 (82.39)		207 (72.38)	276 (83.15)		1290 (71.23)	1900 (82.14)	
**Age group (years)**			0.0422			0.0779			0.1695
18–29	680 (32.43)	760 (28.78)		124 (43.36)	115 (35.06)		556 (30.70)	645 (27.89)	
30–39	826 (39.26)	1093 (41.39)		110 (38.46)	128 (39.02)		716 (39.54)	965 (41.72)	
40–49	447 (21.32)	579 (21.92)		42 (14.69)	68 (20.73)		405 (22.36)	511 (22.09)	
≥50	144 (6.87)	209 (7.91)		10 (3.50)	17 (5.18)		134 (7.40)	192 (8.30)	
**Length of most recent incarceration**			<0.01			<0.01			<0.01
1–4 days	796 (37.96)	264 (10.00)		148 (51.75)	40 (12.20)		648 (35.78)	224 (9.68)	
5–17 days	518 (24.70)	552 (20.90)		73 (25.52)	88 (26.83)		445 (24.57)	464 (20.06)	
18–53 days	490 (23.37)	811 (30.71)		43 (15.03)	106 (32.32)		447 (24.68)	705 (30.48)	
> 54 days	293 (13.97)	1014 (38.39)		22 (7.69)	94 (28.66)		271 (14.96)	920 (39.78)	
**Number of previous provincial incarcerations** ^§^			0.5752			0.5036			0.2740
0	338 (16.12)	415 (15.71)		69 (24.13)	77 (23.48)		269 (14.85)	338 (14.61)	
1	509 (24.27)	676 (25.60)		90 (31.47)	91 (27.74)		419 (23.14)	585 (25.29)	
>2	1250 (59.61)	1550 (58.69)		127 (44.41)	160 (48.78)		1123 (62.01)	1390 (60.10)	
**Mental health diagnosis** ^«^			<0.01			<0.01			
No	1304 (62.18)	2027 (76.75)		145 (50.70)	226 (68.90)		1159 (64.00)	1801 (77.86)	
Yes	793 (37.82)	614 (23.25)		141 (49.30)	102 (31.10)		652 (36.00)	512 (22.14)	
**Number of chronic conditions**			<0.01			0.1470			<0.01
None	984 (46.92)	1508 (57.10)		98 (34.27)	131 (39.94)		886 (48.92)	1377 (59.53)	
One or more	1113 (53.08)	1133 (42.90)		188 (65.73)	197 (60.06)		925 (51.08)	936 (40.47)	
**Year of release**			<0.01			<0.01			<0.01
2015	598 (28.2)	311 (11.78)		79 (27.62)	29 (8.84)		519 (28.66)	282 (12.19)	
2016	597 (28.47)	505 (19.12)		88 (30.77)	68 (20.73)		509 (28.11)	437 (18.89)	
2017	514 (24.51)	873 (33.06)		81 (28.32)	122 (37.20)		433 (23.91)	751 (32.47)	
2018	388 (18.50)	952 (36.05)		38 (13.29)	109 (33.23)		350 (19.33)	843 (36.45)	
**Released after Transfer** ^**⊥**^			<0.01			<0.01			<0.01
No (before)	1579 (75.30)	1468 (55.59)		235 (82.17)	191 (58.23)		1344 (74.21)	1277 (55.21)	
Yes (after)	518 (24.70)	1173 (44.41)		51 (17.83)	137 (41.77)		467 (25.79)	1036 (44.79)	

NOFD = nonfatal overdose OAT = Opioid Agonist Treatment

^**‡**^Chi-square test; p<0.05

^**†**^Record of nonfatal overdose in data from Emergency Health Services (BCEHS), Drug and Poison Information Centre (DPIC), case-based reporting from Emergency Departments, National Ambulatory Care Reporting System (NACRS), Discharge Abstract Database (DAD) or Medical Services Plan (MSP). Fatal overdose identified in Vital Statistics and BC Coroner Records. Nonfatal and fatal overdoses were combined for descriptive statistics as information sharing agreements require suppression of data <5. None of the women who died of overdose received OAT in custody. Among men, there were three more deaths due to overdose among men who did not receive OAT in custody compared to those who did.

^◊^Any record of OAT dispensation in British Columbia between January 1, 2010, and date of admission to custody.

^§^ In British Columbia, between January 1, 2010, and date of release.

^«^Mental Health Diagnosis includes ICD-10 codes classifying mental, behavioural and neurodevelopmental disorders, excluding those related to psychoactive substance use and ICD-9 codes classified as Mental Disorders excluding drug or alcohol-related psychoses, dependence, or non-dependent abuse of drugs (S3 Table).

^**⊥**^Responsibility for healthcare services transferred from a private for-profit company contracted by BC Corrections to the Provincial Health Services Authority on October 1, 2017

In unadjusted analysis receipt of OAT during incarceration was associated with a reduced hazard of nonfatal overdose in the 30 days after release (HR 0.59, 95% CI 0.45,0.77). Increased hazard of nonfatal overdose was associated with previous provincial incarcerations, having one or more chronic health conditions and having a mental health diagnosis (Table 2). In the model adjusted for age, sex, length of most recent incarceration, number of previous provincial incarcerations, mental health diagnosis, number of chronic health conditions and year of release, OAT use during incarceration was associated with a decrease (aHR 0.55, 95% CI 0.41, 0.74) in the hazard of nonfatal overdose in the 30 days after release.

**Table 2:** Unadjusted and adjusted hazard ratios for nonfatal overdose following release from provincial correctional facilities between January 1, 2015, and December 1, 2018 in a 20% random sample of the population of British Columbia.

	Unadjusted (HR 95% CI)	Adjusted (HR 95% CI)
**Received OAT while incarcerated**		
No	Ref	Ref
Yes	0.59 (0.45, 0.77)	0.55 (0.41, 0.74)
**Age group (years)**		
18–29	Ref	Ref
30–39	0.99 (0.67, 1.45)	1.02 (0.71, 1.47)
40–49	0.83 (0.57, 1.21)	0.90 (0.64, 1.29)
≥50	0.87 (0.49, 1.54)	0.95 (0.54, 1.65)
**Sex**		
Male	Ref	Ref
Female	1.17 (0.65, 2.10)	1.10 (0.63, 1.91)
**Length of most recent incarceration**		
1–4 days	Ref	Ref
5–17 days	1.26 (0.86, 1.83)	1.50 (1.02, 2.22)
18–53 days	1.56 (1.07, 2.25)	1.91 (1.31, 2.80)
> 54 days	1.10 (0.76, 1.59)	1.85 (1.26, 273)
**Number of previous provincial incarcerations** ^§^		
0	Ref	Ref
1	1.94 (1.21, 311)	1.70 (1.06, 2.72)
2+	3.72 (2.41, 5.74)	2.40 (1.56, 370)
**Number of chronic conditions**		
0	Ref	Ref
1 or more	2.60 (1.89, 3.57)	1.56 (1.15, 211)
**Mental health diagnosis** ^«^		
No	Ref	Ref
Yes	3.76 (2.87, 4.93)	2.62 (1.98, 3.46)
**Year of release**		
2018	Ref	Ref
2017	1.44 (1.06, 1.95)	1.24 (0.92, 1.68)
2016	1.34 (0.91, 1.98)	1.01 (0.68, 1.50)
2015	0.71 (0.46, 1.11)	0.57 (0.36, 0.90)

OAT = Opioid Agonist Therapy; HR = Hazard Ratio; CI = Confidence Interval

^§^ In British Columbia, between January 1, 2010, and date of release.

^«^Mental Health Diagnosis includes ICD-10 codes classifying mental, behavioural and neurodevelopmental disorders, excluding those related to psychoactive substance use and ICD-9 codes classified as Mental Disorders excluding drug or alcohol-related psychoses, dependence, or non-dependent abuse of drugs (S3 Table).

Compared to people who did not receive OAT in custody, in the unadjusted model a decreased hazard of nonfatal overdose was observed for OAT continued from community (HR 0.49 95%CI 0.36, 0.68) and for OAT episodes initiated in custody (HR 0.66 95% CI 0.48, 0.89). In the adjusted model, compared to those who did not receive OAT, a decreased hazard of nonfatal overdose after release was seen among people who continued OAT from the community (aHR 0.49, 95%CI 0.36, 0.67) and people who initiated a new episode of OAT in custody (aHR 0.58 95%CI 0.41, 0.82; Table 3).

**Table 3:** Unadjusted and adjusted hazard ratios for nonfatal overdose following release from provincial correctional facilities in British Columbia between January 1, 2015 and December 1, 2018 among people who did not receive OAT, people who continued a community prescription and people who initiated a new episode of OAT in custody.

	Unadjusted (HR 95% CI)	Adjusted (HR 95% CI)
**OAT while incarcerated**		
No OAT	Ref	Ref
Continued OAT	0.49 (0.36, 0.68)	0.49 (0.36,0.67)
New episode of OAT	0.66 (0.48, 0.89)	0.58 (0.41, 0.82)
**Age group (years) at time of release**		
18–29	Ref	Ref
30–39	0.99 (0.67, 1.45)	1.02 (0.72, 1.46)
40–49	0.83 (0.57, 1.21)	0.98 (0.69, 1.39)
≥50	0.87 (0.49, 1.54)	0.96 (0.55, 1.66)
**Sex**		
Male	Ref	Ref
Female	1.17 (0.65, 2.10)	1.08 (0.62, 1.85)
**Length of most recent incarceration**		
1–4 days	Ref	Ref
5–17 days	1.26 (0.86, 1.83)	1.49 (1.02, 2.19)
18–53 days	1.56 (1.07, 2.25)	1.94 (1.33, 2.84)
> 54 days	1.10 (0.76, 1.59)	1.91 (1.30, 2.81)
**Number of previous provincial incarcerations** ^§^		
0	Ref	Ref
1	1.94 (1.21, 311)	1.66 (1.06, 2.59)
2+	3.72 (2.41, 5.74)	2.24 (1.49, 3.39)
**Number of chronic conditions**		
0	Ref	Ref
1 or more	2.60 (1.89, 3.57)	1.53 (1.14, 2.06)
**Mental health diagnosis** ^«^		
No	Ref	Ref
Yes	3.76 (2.87, 4.93)	2.56 (1.95, 3.36)
**Year of release**		
2018	Ref	Ref
2017	1.44 (1.06, 1.95)	1.27 (0.94, 1.71)
2016	1.34 (0.91, 1.98)	1.05 (0.71, 1.56)
2015	0.71 (0.46, 1.11)	0.58 (0.36, 0.92)

OAT = Opioid Agonist Therapy; HR = Hazard Ratio; CI = Confidence Interval

^§^ In British Columbia, between January 1, 2010 and date of release.

^«^Mental health diagnosis includes ICD-10 codes classifying mental, behavioural and neurodevelopmental disorders, excluding those related to psychoactive substance use and ICD-9 codes classified as Mental Disorders excluding drug or alcohol-related psychoses, dependence, or non-dependent abuse of drugs (S3 Table)

### Subgroup analyses

The association between OAT use during incarceration and nonfatal overdose after release was observed for both women and men in subgroup analysis stratified by sex. Among releases of women, OAT dispensation during incarceration was associated with a decreased hazard of nonfatal overdose in unadjusted (HR 0.55, 95%CI 0.32, 0.96) and adjusted analysis (aHR 0.29 95% CI 0.14, 0.58; Table 4). In a second adjusted model, compared to women who did not receive OAT, a reduced hazard of nonfatal overdose after release was seen among women who initiated a new episode of OAT in custody (aHR 0.16 95%CI 0.053, 0.46) and women who continued treatment from the community (aHR 0.49 95% CI 0.27, 0.88).

**Table 4:** Unadjusted and adjusted hazard ratios of nonfatal overdose after release from provincial correctional facilities between January 1, 2015 and December 1, 2018 among women and men in a 20% random sample of the population of British Columbia.

	Women		Men	
	Unadjusted HR (95%CI)	Adjusted HR (95%CI)	Unadjusted HR (95%CI)	Adjusted HR (95%CI)
**Received OAT while incarcerated**				
No	Ref	Ref	Ref	Ref
Yes	0.55 (0.32, 0.96)	0.29 (0.14, 0.58)	0.60 (0.45, 0.80)	0.60 (0.45, 0.81)
**Age group (years)**				
18–29	Ref	Ref	Ref	Ref
30–39	1.78 (0.55, 5.79)	1.74 (0.63, 4.80)	0.89 (0.60, 1.31)	0.92 (0.65, 1.32)
40–49	1.63 (0.69, 3.87)	1.58 (0.77, 3.26)	0.74 (0.49, 1.12)	0.84 (0.57, 1.24)
≥50	1.41 (0.29, 6.74)	2.17 (0.52, 9.03)	0.81 (0.44, 1.50)	0.85 (0.47, 1.52)
**Length of most recent incarceration**				
1–4 days	Ref	Ref	Ref	Ref
5–17 days	2.07 (0.75, 5.71)	2.59 (0.97, 6.96)	1.12 (0.76, 1.65)	1.30 (0.90, 1.90)
18–53 days	2.00 (0.65, 6.16)	2.74 (0.82, 9.09)	1.49 (1.00, 2.21)	1.76 (1.23, 2.51)
> 54 days	1.53 (0.64, 3.64)	4.15 (1.43, 12.07)	1.04 (0.69, 1.57)	1.65 (1.12, 2.44)
**Number of previous provincial incarcerations** ^§^				
0	Ref	Ref	Ref	Ref
1	1.62 (0.79, 3.34)	1.20 (0.54, 2.67)	2.15 (1.18, 3.94)	1.94 (1.06, 3.55)
>2	2.58 (1.32, 5.04)	1.59 (0.70, 3.61)	4.40 (2.53, 7.64)	2.78 (1.60, 4.82)
**Mental health diagnosis** ^«^				
No	Ref	Ref	Ref	Ref
Yes	2.60 (1.52, 4.45)	1.87 (1.06, 3.30)	4.00 (2.95, 5.43)	2.75 (2.02, 3.75)
**Number of chronic conditions**				
None	Ref	Ref	Ref	Ref
One or more	2.47 (0.99, 6.19)	1.94 (0.89, 4.24)	2.62 (1.88, 3.64)	1.52 (1.11, 2.08)
**Year of release**				
2018	Ref	Ref	Ref	Ref
2017	1.00 (0.61, 1.66)	0.72 (0.42, 1.24)	1.53 (1.07, 2.18)	1.34 (0.95, 1.88)
2016	0.82 (0.30, 2.20)	0.50 (0.20, 1.26)	1.47 (0.97, 2.22)	1.13 (0.75, 1.70)
2015	0.27 (0.08, 0.93)	0.13 (0.03, 0.51)	0.82 (0.51, 1.31)	0.69 (0.43, 1.10)

OAT = Opioid Agonist Therapy; HR = Hazard Ratio; CI = Confidence Interval

^§^ In British Columbia, between January 1, 2010 and date of release.

^«^Mental health diagnosis includes ICD-10 codes classifying mental, behavioural and neurodevelopmental disorders, excluding those related to psychoactive substance use and ICD-9 codes classified as Mental Disorders excluding drug or alcohol-related psychoses, dependence, or non-dependent abuse of drugs (S3 Table)

Among men, receipt of OAT in custody was associated with reduced hazard of nonfatal overdose after release in unadjusted analysis (HR 0.60 95%CI 0.45, 0.80), and adjusted analysis (aHR 0.60 95%CI 0.45, 0.81; Table 4). Compared to men who did not receive OAT in custody, a reduced hazard of nonfatal overdose after release was seen for men who initiated a new episode of OAT in custody (aHR 0.70, 95%CI 0.51, 0.98), and for men who were continuing a community OAT prescription (aHR 0.47 95% CI 0.33, 0.70).

### Sensitivity analyses

There were 59 incarceration events where OAT was only dispensed on the date of admission. Since OAT dispensed on the day of admission could have been dispensed in custody or in the community prior to arrest, as a sensitivity analysis we considered only incarceration events where OAT was dispensed after the date of admission as having received OAT while incarcerated. This change did not affect the estimate in unadjusted (HR 0.60, 95% CI 0.46, 0.79) or adjusted analyses (aHR 0.55, 95% CI 0.41, 0.74). As a second sensitivity analysis we replaced the variable for year of release with a dichotomous variable of whether the release occurred before or after the transfer of healthcare services to the Ministry of Health (October 1, 2017). This produced a similar estimate (aHR 0.62, 95%CI 0.47, 0.82). We also examined the relationship between receipt of OAT in custody and any overdose (fatal or nonfatal) in the first 30 days after release. Similar to estimates for nonfatal overdose, receipt of OAT in custody was associated with reduced hazard of overdose in unadjusted (HR 0.59, 95%CI 0.46, 0.77) and adjusted (aHR 0.54, 95%CI 0.41, 0.72) models.

To better understand the effect on people initiating OAT for the first time while in custody we examined the association between OAT during incarceration and nonfatal overdose after release separating those with and without a prior history of use of OAT. Compared to people who did not receive OAT, use of OAT in custody was associated with a decreased hazard of nonfatal overdose after release among people initiating OAT for the first time (aHR 0.40, 95% CI 0.211, 0.77), people initiating a new episode of OAT in custody who had a previous history of OAT use (aHR 0.64 95%CI 0.44, 0.92) and people continuing a community prescription (aHR 0.50 95% CI 0.36, 0.68).

Poisson regression produced similar estimates to the main model in unadjusted (RR 0.58, 95%CI 0.45,0.76) and adjusted (aRR 0.56, 95% CI 0.42, 0.75) analyses, as did negative binomial regression (RR 0.43, 95% CI 0.33, 0.56; aRR 0.43, 95%CI 0.33, 0.58). Due to the small sample of women, we conducted a sensitivity analysis including an interaction term for sex and use of OAT in custody. Compared to women who did not receive OAT, men who received OAT (aHR 0.47 95% CI 0.23, 0.98) and women who received OAT (0.44 95% CI 0.25, 0.79) had a reduced hazard of nonfatal overdose after release.

## Discussion

We found that receipt of OAT during incarceration was associated with a reduced hazard of nonfatal overdose in the 30 days following release from provincial correctional facilities in BC. The protective effect of OAT during incarceration was observed for people who continued a community prescription and for people who initiated a new episode of OAT in custody. We also found that OAT receipt during incarceration significantly reduced the hazard of nonfatal overdose after release among both women and men, but that the effect seen was larger among women.

In our study, only 40% of people who received OAT while in custody had an active community prescription in the week prior to their incarceration and 18% had no prior history of OAT use. In many jurisdictions across Canada and the US, if OAT is available in custody it is limited to people with a current community prescription [3, 4] indicating a high level of unmet need. Expanding access to OAT in custody may require addressing both policy-level and practice-level barriers. For example, providers in Ontario identified multiple systemic barriers to initiating OAT in provincial correctional facilities including lack of resources and the absence of links to community providers [55]. In BC, expansion of access to OAT in custody came from a number of policy and legal changes including buprenorphine/naloxone becoming a regular benefit under the provincial PharmaCare program [56]. In 2016 the province declared a public health emergency of overdose deaths [57] which garnered political will and funding to prevent and address harms from the toxic drug supply. Also in 2016, BC Corrections settled a charter challenge about access to OAT in provincial correctional facilities [58]. Additionally, the transfer of responsibility for healthcare services in BC provincial correctional facilities to the Ministry of Health included an explicit focus on improving services for mental health and substance use [47, 59]. We found a higher proportion of people released after the transfer received OAT in custody which is consistent with reports of improved access since the transfer to the Ministry of Health, including elimination of the waitlist for OAT in provincial custody [48, 59]. In June 2023, BC introduced full coverage for OAT under the province’s universal health insurance plan (MSP) [60]. Removing financial barriers to access in the community may support continuity of use after release. Custodial settings offer key opportunities to initiate or reinitiate OAT [61], but should be integrated within a continuum of care including appropriate release planning, community health linkages and supports.

In this study, people with an active community prescription or who had a history of OAT use prior to arrest were more likely to have received OAT during their incarceration. People who received OAT in custody were less likely to have a mental health diagnosis or a chronic health condition. This is consistent with a study in Veterans Health Administration facilities [62] which found that people with concurrent mental health diagnoses were less likely to access OAT. Studies have shown a high prevalence of co-occurring mental health diagnoses among people with substance use disorders in prisons [63] and in nonincarcerated populations [64]. Research is needed to understand, and respond to, this disparity in access which may reflect both institutional policies and barriers and individual patients’ knowledge, preferences, and experiences [65, 66].

OAT receipt during incarceration was associated with a large reduction in the hazard of nonfatal overdose after release among women. Increased accessibility to OAT for incarcerated women, particularly for women who do not have an active community OAT prescription, may help to reduce harms during the acute period of risk following release. Increasing access for women requires specific, targeted approaches. On average, women spend less time in remand and sentenced custody [67] which may affect access or stability of OAT treatment. Furthermore, women face greater stigma related to drug use and more often experience violence, homelessness or loss of custody of children as a result, which may act as a barrier to care [31]. Further research is needed to inform tailored, gender-responsive programming to increase OAT access during incarceration and after release.

Finally, in 44% releases of people with OUD, the person did not receive OAT during their incarceration (including 144 incarceration episodes in which the person had an active community prescription prior to admission). Future research should examine barriers and opportunities to accessing OAT in custody, as well as alternative treatments and supports for people who do not want to use OAT or for whom OAT is insufficient. These may include expansion of access to harm-reduction services and other supports such as supervised consumption sites, drug testing and a safer (pharmaceutical grade) supply [68, 69].

This study had several strengths. We used a large, representative sample of the BC population which supports the generalizability of findings to BC and similar populations. Furthermore, administrative records allowed us to establish the temporality of the relationship between OAT use in custody and nonfatal overdose after release. Sensitivity analyses for the definition of receipt of OAT in custody, for the change in healthcare governance and that included fatal overdose in the outcome variable, all produced similar estimates. Additionally, sensitivity analysis for people without a history of OAT use demonstrate the effect across categories of previous exposure. Finally, both Poisson and negative binomial models produced estimates similar to the primary analysis indicating robustness in our findings. This study also had several limitations. One limitation of our study pertains to the exploration and interpretation of interaction effects, especially those involving the non-modifiable factor of sex. The intrinsic, non-modifiable nature of sex introduces complexities when assessing confounding in an observational setting, particularly since confounders cannot alter such a factor. Although interactions on a multiplicative scale can provide valuable statistical information, they may not always translate into clear public health or clinical interventions, especially when they involve non-modifiable attributes [70]. These interpretational challenges, combined with our aim to provide specific insights into the differential impacts of OAT within distinct sex categories, prompted us to stratify our analyses by sex. To provide additional context and depth to our primary findings without attributing undue weight to them, we opted to present interaction analyses as a sensitivity analysis. This approach acknowledges the potential challenges and pitfalls that can arise from interpreting interaction effects in observational studies. Nevertheless, we recognize the importance of thoroughly exploring and presenting interaction effects and encourage future researchers to consider incorporating additive interaction analyses where they align with the research question and design.

Additionally, overdoses reversed in the community where healthcare was not called or where the person was not on scene when the paramedics arrived are not captured in administrative data and so nonfatal overdoses are underreported in our study. The number of fatal overdose events captured in our data was not sufficient to allow comparison of fatal and nonfatal overdoses. Future research is needed using a larger population or longer time period. Administrative data also has limitations on the specificity of identifying people with OUD. In this study we included only people who had diagnostic codes that specified opioid use. We protected against misclassification by requiring at least two physician billing records for OUD. In October of 2015, buprenorphine/naloxone became a regular benefit under Pharmacare, BC’s drug insurance plan [71]. Kurz and colleagues found a subsequent increase in use of OAT in custody, largely driven by increased use of buprenorphine/naloxone among people who were not using OAT at the time of their admission [72]. For this reason we did not examine outcomes by type of OAT prescribed, however future research should examine potential differences in outcomes and risks related to nonfatal overdose specific to OAT modalities delivered in custody. In this analysis, we were unable to explore the impact of choice in the use of OAT or in outcomes, which may have affected estimates. Due to limitations in how prescription data is recorded we could not assess whether people received OAT in the days immediately preceding release. We did not assess continuity of OAT prescription after release from custody. Previous research indicates an increased mortality risk following cessation [25, 73] or disruption [23] of OAT. Furthermore, studies have found that maintaining OAT after release is associated with reduced mortality [25] and fewer encounters with emergency medical services [74] compared to people who experienced interruptions or did not use OAT. Future research should examine how continuity of OAT after release from custody affects the risk of nonfatal overdose. No information on race, ethnicity or Indigenous identity was available for use in this study. Finally, this study focused on outcomes in the short time after release when risk of overdose is highest [14, 15, 18, 21, 22]. Research is needed to understand the long-term outcomes of expanding access to OAT in correctional settings, particularly for people initiating a new episode of OAT while incarcerated.

We found that OAT receipt during incarceration was associated with decreased hazard of nonfatal overdose in the weeks following release from custody and that the effect was larger among women. Efforts are needed to ensure stable access to OAT throughout incarceration and that supports for initiation and maintenance of OAT in custody are gender-responsive. Our study found that OAT provision in custody is protective against non-fatal overdose for individuals initiating new OAT episodes. Correctional facilities should be leveraged to initiate care, while ensuring policies and support structures are in place for seamless continuity after release.

## Supporting information

S1 Table Description of datasets in the British Columbia Provincial Overdose Cohort used in this study. (DOCX)

S2 Table Case definition of overdose used in the British Columbia Provincial Overdose Cohort. (DOCX)

S3 Table ICD-9 and ICD-10 codes used for identification of Opioid Use Disorder and mental health diagnoses in records from medical services plan and discharge abstract database. (DOCX)
