# Supplementary material for: Receipt of Opioid Agonist Treatment in provincial correctional facilities in British Columbia is associated with a reduced hazard of nonfatal overdose in the month following release
Source: PLoS One. 2024 Jul 10;19(7):e0306075. doi: 10.1371/journal.pone.0306075 (PMC11236203; doi:10.1371/journal.pone.0306075)
Supplement: S1 Table — (DOCX) [file pone.0306075.s001.docx]

**S1 Table. Description of datasets in the British Columbia Provincial Overdose Cohort used in this study**

| **Dataset** | **Description** |
| --- | --- |
| Ministry of Health Provincial Client Roster | Contains demographic characteristics, death date, and location information of BC residents for each calendar year available after 2010. |
| BC Emergency Health Services (BCEHS) | Contains information about the time and location of an overdose event, demographic information about patients, and details from the dispatch and paramedic’s assessment, treatment, and transportation of patients. |
| Drug and Poison Information Centre (DPIC) | Contains information about calls to the DPIC from the public or medical personnel for advice on poisoning management. |
| BC Coroners Service (BCCS) | BCCS investigates all unnatural, sudden and unexpected, unexplained or unattended deaths in the province including all accidental and undetermined illicit drug-related overdose deaths.[46] |
| Enhanced Emergency Department (EED) records | Contains data from paper-based reporting of opioid- and drug-related overdose in emergency departments in three of the five BC Health Authorities. |
| National Ambulatory Care Reporting System (NACRS) | NACRS is a national database designed to capture information on patient visits to hospital-based and community-based ambulatory care. |
| Discharge Abstract Database (DAD) | Contains discharges, transfers, and deaths occurring in acute care hospitals in BC. |
| Medical Services Plan (MSP) | Contains records of all fee-for-service physician visits billed to the province’s universal health insurance program. |
| PharmaNet | Contains records of all ambulatory care prescription dispensations in the province of BC. |
| BC Corrections | Includes demographic, admission, transfer, and discharge information of adults (aged 18 years and older) in BC provincial correctional facilities. |
| Vital Statistics | Vital Statistics captures cause of death information from residents of BC who have died in BC. |
