# Supplementary material for: Receipt of Opioid Agonist Treatment in provincial correctional facilities in British Columbia is associated with a reduced hazard of nonfatal overdose in the month following release
Source: PLoS One. 2024 Jul 10;19(7):e0306075. doi: 10.1371/journal.pone.0306075 (PMC11236203; doi:10.1371/journal.pone.0306075)
Supplement: S2 Table — (DOCX) [file pone.0306075.s002.docx]

**S2 Table. Case definition of overdose used in the British Columbia Provincial Overdose Cohort**

| **Dataset** | **Overdose Definition** |
| --- | --- |
| BC Emergency Health Services (BCEHS) | Naloxone administered by paramedics, or paramedic impression code for: recreational drug overdose AND cardiac or respiratory arrest/death, overdose/poisoning (ingestion), sick, unconscious, or impression code for: opioid related, opioid related / overdose or cardiac arrest AND overdose/poisoning (ingestion).^35^ |
| Drug and Poison Information Centre (DPIC) | A record indicative of opioid consumption (37701–05, 37707–8, 37784, 41700, 72700, 72702, 72704, 77810, 200625, 200628, 200630, 200638, 201063, 201131) |
| Enhanced Emergency Department (EED) records | Physician assessment that clinical symptoms indicate an opioid overdose |
| Medical Services Plan (MSP) | ICD-9 code of 965.0 or E850 |
| Discharge Abstract Database (DAD) | Primary discharge diagnosis ICD-10 code of T40.0-T40.6 |
| National Ambulatory Care Reporting System (NACRS) | ICD-10 code of T40.1 or T40.6 in the emergency department discharge diagnosis field |
| BC Coroners Service (BCCS) | Illicit drug toxicity includes street drugs (heroin, fentanyl, cocaine, MDMA, methamphetamine, etc.), medications that were not prescribed to the deceased person, combinations of the above, with prescribed medications, and those overdoses where the origin of drug is not known. Includes open investigations (toxicology pending) and closed drug toxicity death |
| Vital Statistics Deaths | ICD-10 cause-of-death codes X40–X44, X60–X64, X85, and Y10–Y14 |

ICD-10/ICD-9 = International classification of disease 10^th^ edition/9^th^ edition
