# Supplementary material for: Receipt of Opioid Agonist Treatment in provincial correctional facilities in British Columbia is associated with a reduced hazard of nonfatal overdose in the month following release
Source: PLoS One. 2024 Jul 10;19(7):e0306075. doi: 10.1371/journal.pone.0306075 (PMC11236203; doi:10.1371/journal.pone.0306075)
Supplement: S3 Table — (DOCX) [file pone.0306075.s003.docx]

**S3 Table. ICD-9 and ICD-10 codes used for identification of Opioid Use Disorder and mental health diagnoses in records from Medical Services Plan and Discharge Abstract Database.**

| **Description** | **Code** |
| --- | --- |
| ICD-10 codes for OUD | F11 |
| ICD-9 codes for OUD | 304.0, 304.7, 305.5 |
| ICD 10-codes for mental health diagnosis | F01-F09, F20-F99 |
| ICD-9 Codes for mental health diagnosis | 290, 293 – 302, 306-319, 50B |

ICD-10/ICD-9 = International classification of disease 10^th^ edition/9^th^ edition

OUD = Opioid Use Disorder
